# Supplementary material for: Intranasal and Serum Gentamicin Concentration: Comparison of Three Topical Administration Protocols in Dogs
Source: Vet Sci. 2023 Jul 28;10(8):490. doi: 10.3390/vetsci10080490 (PMC10457901; doi:10.3390/vetsci10080490)
Supplement: Supplementary file 1 [file vetsci-10-00490-s001.zip › vetsci-2487954-supplementary.pdf]

VIDEO S1: Procedure of nasal lavage. A healthy beagle dog was placed in ventral recumbency. The nasopharynx was manually obstructed. A 4 cm-long 12 Fr fenestrated catheter connected to a 60 mL syringe was introduced in the first third of left nasal cavity. Twenty mL (± 1 mL/kg) of sterile isotonic saline solution were injected with the ipsilateral nostril being manually obstructed to prevent leakage of the solution. The fluid was then directly aspirated in the syringe using a manual suction while progressively removing the catheter from the nasal cavity.

Video supporting information.mp4
